# Molecular insights into probiotic mechanisms of action employed against intestinal pathogenic bacteria

**DOI:** 10.1080/19490976.2020.1831339

**Published:** 2020-10-28

**Authors:** Winschau F. van Zyl, Shelly M. Deane, Leon M.T. Dicks

**Affiliations:** Department of Microbiology, Stellenbosch University, Stellenbosch, South Africa

**Keywords:** Probiotics, lactic acid bacteria, colonization, gastrointestinal tract, enteric pathogens, competitive exclusion, antimicrobial compounds, bacteriocins

## Abstract

Gastrointestinal (GI) diseases, and in particular those caused by bacterial infections, are a major cause of morbidity and mortality worldwide. Treatment is becoming increasingly difficult due to the increase in number of species that have developed resistance to antibiotics. Probiotic lactic acid bacteria (LAB) have considerable potential as alternatives to antibiotics, both in prophylactic and therapeutic applications. Several studies have documented a reduction, or prevention, of GI diseases by probiotic bacteria. Since the activities of probiotic bacteria are closely linked with conditions in the host’s GI-tract (GIT) and changes in the population of enteric microorganisms, a deeper understanding of gut-microbial interactions is required in the selection of the most suitable probiotic. This necessitates a deeper understanding of the molecular capabilities of probiotic bacteria. In this review, we explore how probiotic microorganisms interact with enteric pathogens in the GIT. The significance of probiotic colonization and persistence in the GIT is also addressed.

## Introduction

Lactic acid bacteria (LAB) play a major role in the preservation and organoleptic profile of fermented food products, but are equally important in affecting the composition and diversity of intestinal microbiota.^1–3^ Some of the most important beneficial effects include stimulation of the host’s immune system, prevention of antibiotic-associated diarrhea, treatment of inflammatory bowel disease (IBD) and irritable bowel syndrome (IBS), alleviation of lactose intolerance, lowering of cholesterol levels and prevention of life-threatening GI infections such as *Clostridium difficile*-associated diarrhea.^4–8^ Renewed interest in probiotics initiated the launch of an increasing number of probiotic-containing supplements that claim to confer specific health benefits to the consumer.^9,10^ Many of these products are driven by aggressive marketing through pharmaceutical and nutritional companies, often without a clear understanding of the interactions between probiotic bacteria, normal commensal microorganisms, pathogens and the host.

Lactic acid bacteria are indigenous to the small and large intestine of humans and animals and exert a number of probiotic properties, such as binding to receptors and physically excluding pathogens, production of antimicrobial substances, strengthening of the gut mucosal barrier and modulation of the immune system.^11–15^ It is therefore important to have an in-depth understanding of the specific metabolic and genetic interactions between probiotic bacteria, the host intestinal mucosa and enteric pathogens in the GIT. Commensal bacteria also act as a protective barrier against pathogens by providing mucosal protection and stimulation of the immune system.^10,15^ The most predominant genera used in probiotic supplements are *Lactococcus, Lactobacillus* and *Bifidobacterium* spp. derived from humans and animals.^11^ It is important that probiotic strains survive passage through the stomach, resist bile salts and digestive enzymes in the small intestinal tract and reach the colon in sufficient numbers.^16^ The number of viable cells surviving the journey through the GIT is, however, strain specific and depends on the dosage and duration of administration.^10,16^

Due to the complex nature of the human GIT, only a few in-depth studies on interactions between probiotic bacteria and enteric pathogens have been published and many rely on *in vitro* data to decipher the mechanistic basis underlying a specific health benefit.^17^ A specific health benefit may also be attributed to a combination of mechanisms. This is important, as strains from the same species may evoke different responses in the host.^13^ Several probiotic strains secrete secondary metabolites and peptides with antimicrobial activity that may interact directly with the host or pathogens.^15,18^ Ultimately, a detailed characterization of these interactions will significantly improve the application of probiotics to support and enhance human health. In this review, the mechanisms probiotic bacteria use to interact with enteric pathogens, and the ability to colonize the GIT, are discussed.

## Colonization and persistence of probiotic bacteria in the GIT

Strains that colonize the GIT have a greater beneficial effect on the host than strains passing through the GIT.^19–21^ This may be because adhesion to mucus and epithelial cells not only provides the strain with a competitive advantage, but forms a stronger interaction with the host that leads to recognition of the probiotic and stimulation of the host’s immune response.^15,17^ Furthermore, colonization by probiotic strains prevents adhesion of enteric pathogens to intestinal cells.^22^ Several studies have shown how probiotics interfere with the ability of GI pathogens, such as *Salmonella typhimurium, Clostridium sporogenes* and *Enterococcus faecalis*, to adhere to Caco-2 cells.^23–25^ A diverse spectrum of pathogens targeted by probiotic bacteria and their reported health promoting effects is listed in Table 1.

**Table 1.** Health effects of probiotic bacteria and main pathogens targeted

Probiotic strains	Pathogen(s)^a^	Reported effects
*Lactobacillus rhamnosus* GG ^4,26,27^	*Helicobacter pylori, rotavirus, C. difficile*	Reduced diarrhea and nausea in a human trial. Immune enhancement. Used for alleviation of atopic dermatitis in children, stabilization of intestinal permeability
*L. johnsonii* La1 ^28^	*H. pylori*	Regular ingestion modulated *H. pylori* colonization in children
*L. casei* DG ^29^	*H. pylori*	Increased eradication rate of *H. pylori* infection when supplemented with first-line therapies
*L. casei* CRL431 ^30^	*Salmonella enterica* serovar Typhimurium	Preventative administration protected mice against infection
*L. rhamnosus* HN001 ^31^	*Salmonella enterica* serovar Typhimurium	Conferred immune enhancement and protection against *Salmonella* infection in mice
*Bifidobacterium longum* Bb46 ^32^	*Salmonella enterica* serovar Typhimurium	Protective effect against *Salmonella* challenge in gnotobiotic mice
*L. plantarum* 423 and *Enterococcus mundtii* ST4SA ^33^	*Salmonella enterica* serovar Typhimurium	Alleviated symptoms of *Salmonella* infections in challenge study using rats
*L. casei* BL23 and *L. paracasei* CNCM I-3689 ^34^	*Listeria monocytogenes*	Decreased pathogen systemic dissemination in orally infected mice
*L. salivarus* UC118 ^35^	*L. monocytogenes*	Protected mice from pathogenic infection in liver and spleen
*L. plantarum* 423 and *E. mundtii* ST4SA ^36,37^	*L. monocytogenes*	Excluded the pathogen from the intestinal tract of mice after daily administrations of probiotic strains
*Lactococcus lactis* MM19 and *Pediocin acidilactici* MM33 ^38^	Vancomycin resistant enterococci (VRE)	Modulated intestinal microbiota and reduced pathogen intestinal colonization in mice.
*L. rhamnosus* R0011 and *L. acidophilus* R0052 ^39^	*Citrobacter rodentium*	Pre-treatment with the probiotic strains attenuated pathogen infection in mice
*L. reuteri* ^40^	*C. rodentium*	Attenuated *C. rodentium*-induced colitis in mice. Significantly decreased diarrhea symptoms in infants and children.
*B. breve* ^41^	*Escherichia coli* O157:H7	Protected mice from Shiga toxic-producing *E. coli.*
*Pediococcus pentasaceus* NB-17 ^42^	*n/a*	Effectively stimulated immune cell activities and allergic inhibitory effects
*Oenococcus oeni* 9115 ^43^	*n/a*	Significantly decreased acid-induced colitis in mice. Modulated the immune response of immunocompetent cells *in vitro*.
*B. infantis* UCC 36524 ^4,10,42,43^	*Clostridium*	Reduced clostridia levels and increased lactobacilli and bifidobacteria. Increased blood phagocytic activity. Reduced inflammation in mice.
*Saccharomyces boulardii* ^44,45^	*C. difficile*	Used for prevention and treatment of antibiotics associated and acute diarrhea in children, treatment of *C. difficile* colitis, prevention of diarrhea in critically ill tube-fed patients
*B. adolescentis* ^46^	*Bacteroides thetaiotaomicron*	Significantly modulated both systemic and intestinal immune response in germ-free rats.
*L. acidophilus* ^47^	*n/a*	Reduced the severity of Irritable Bowel Syndrome.

**^a^ Pathogen (s**): n/a, not applicable

Earlier studies on the colonization of probiotics were based on *in vitro* studies demonstrating the ability of strains to adhere to cell lines such as Caco-2, HT-29 and HT29-MTX.^48,49^ Although these studies simulated GIT-models and have provided valuable insights into the adherence of probiotic cells, it remains an *in vitro* approach that is unable to recapitulate the complex multicellular nature of the GIT. Studies using cell lines require specialized equipment and facilities to keep the cells viable. Because of these reasons, studies on the survival and colonization of probiotic bacteria are mostly done by analyzing fecal samples.^50^ From the recovery of cells in feces after probiotic intervention, the persistence of strains is calculated, providing that cell numbers in the dosage are known and all methods are standardized. Probiotic cells that persist in feces for the longest time and highest numbers indicate a higher colonization and persistence in the GIT. *In vivo* pharmacokinetics of probiotics can be studied by comparing cell numbers (in fecal material) between specific strains before and after ingestion.^51,52^ Other techniques used include intestinal intubation and pyxigraphy.^52^ Antibiotic resistance markers can be used to clearly identify probiotic cells in fecal samples.^36,52^ When no strain identification is used, results may be difficult to interpret, since endogenous probiotic cells can also be excreted in feces. The pharmacokinetics of different probiotic LAB using fecal recuperation are listed in Table 2. The survival and persistence of ingested probiotics differs greatly between genera and even between strains. *Lactobacillus* and *Bifidobacterium* spp. have been extensively explored as probiotics, since they form an integral part of the natural gut microbiome of humans and animals.^11,64^ *Bifidobacterium lactis* LAFTI B94, *B. longum* SB T2928, *Lactobacillus rhamnosus* DR20, *Lactobacillus gasseri* SBT2055 and *Enterococcus mundtii* ST4SA persisted in high numbers for the longest time Table 2. In comparison, fecal recuperation of *Lactococcus lactis* MG 1363 and *Lactobacillus fermentum* KLD was much lower. In a recent study, the probiotic strains *Lactobacillus plantarum* 423 and *E. mundtii* ST4SA were transformed with a plasmid containing the bioluminescence firefly luciferase gene (*ffluc*) from *Photinus pyralis*.^59^ This allowed monitoring of the migration of the strains through the GIT and in mouse feces in real time and in a noninvasive manner. With the use of bioluminescent imaging (BLI), the authors detected cell numbers as low as 10^4^ CFU/100 mg feces. Imaging revealed that *E. mundtii* ST4SA persisted in feces throughout the trial period (>20 days), whilst *L. plantarum* 423 persisted for 13 days after the last day (day 5) of intragastric administration.^59^ BLI provides three-dimensional images of cells as they migrate through the GIT (65). Information is gathered in real-time, using an *in vivo* imaging system (IVIS). Only metabolically active cells are detected. The technique has been used in several studies.^36,37,59,65,66^ Van Zyl et al.^59^ used BLI to study the transit of *L. plantarum* 423 and *E. mundtii* ST4SA in the digestive tract of mice for 9 consecutive days. Data generated using the technique correlated with viable cell counts. For a review on the application of optical imaging systems in *in vivo* tracking of LAB, the reader is referred to ref. 66.

**Table 2.** Pharmacokinetics of probiotic strains measured using fecal recuperation

Strain	Dosage	Fecal recuperation	Persistence (day)
*B. lactis* LAFTI B94 ^53^	1 x 10^11^ CFU for 7 days	1.8 x 10^9^ CFU/g	28
*B. lactis* Bb12 ^54^	1 x 10^11^ CFU	8 x 10^7^ CFU/g	14
*B. longum* SB T2928 ^55^	7 x 10^11^ CFU for 7 days	1 x 10^9^ CFU/g	>30
*L. rhamnosus* GG ^56^	6 x 10^10^ CFU for 12 days	4 x 10^4^ CFU/g	14
*L. rhamnosus* DR20 ^57^	1.6 x 10^9^ CFU for ±182 days	6.3 x 10^5^ CFU/g	60
*L. salivarus* UCC118 ^58^	1 x 10^10^ CFU for 21 days	1 x 10^3^–1 × 10^7^ CFU/g	>21
*L. plantarum* 423 ^59^	4 x 10^9^ CFU for 5 days	1 x 10^5^ CFU/g	18 days
*L. plantarum* 299 v ^60^	2 x 10^10^ CFU for 21 days	1 x 10^7^ CFU/g	>8
*L. plantarum* NCIMB 8826 ^51^	5 x 10^10^ CFU for 7 days	1 x 10^8^ CFU/g	14
*L. fermentum* KLD ^51^	1.5 x 10^9^ CFU	3.2 x 10^4^ CFU/g	1
*L. gasseri* SBT2055 ^61^	1 x 10^11^ CFU for 7 days	1 x 10^7^ CFU/g	>31
*Enterococcus mundtii* ST4SA ^59^	4 x 10^9^ CFU for 5 days	1 x 10^6^ CFU/100 mg	>20
*S. thermophilus* ^62^	1.2 x 10^12^ CFU	5 x 10^6^ CFU/g	6
*Lc. lactis* MG 1363 ^63^	1 x 10^11^ CFU for 4 days	1 x 10^4^ CFU/g	3

Some reports have suggested that non-viable and non-colonizing probiotics may also confer certain health benefits to the host.^11,18,67–69^ During GI passage, non-colonizing, or transiently colonizing probiotic bacteria continue to be metabolically active, thus conferring beneficial health effects to their host.^16^ In a study by Kullen et al.^70^ human volunteers were administered a probiotic strain of *Bifidobacterium* and the recovery of the strain in feces was monitored. The strain was detected in feces at increasing cell numbers during days (8 days) of administration, but could not be recovered in fecal material after the last oral administration. The authors concluded that although the administered strain did not colonize the human GIT, colonization and prolonged persistence may not be required to achieve a significant probiotic effect. Similar results were reported by Fujiwara and coworkers.^71,72^ The authors found that bifidobacteria produce a 100 kDa protein, which actively prevents the adherence of pathogenic *Escherichia coli* to intestinal mucosal cells by blocking their binding to the glycolipid binding receptor gangliotetraosylceramide. Therefore, the competitive exclusion of the pathogenic strain may not have been related to direct live cell-to-cell competition for intestinal adhesion sites.

Microorganisms found in fecal samples are usually inhabitants of the lower intestine, such as the colon.^10^ In humans, intubation at specific intestinal sites is used to determine probiotic colonization in the upper sections of the GIT. Biopsies can be taken of the portions of the intestinal tract where probiotics are likely to colonize, proliferate and produce their metabolites.^51,69,73,74^ *Lactobacillus rhamnosus* GG is one of the best studied probiotic strains and plays a role in the prevention or treatment of antibiotic-associated diarrhea, flatulence, rotavirus gastro-enteritis, and stomach and abdominal pain.^26,27,71,72^ However, when *L. rhamnosus* GG in fermented milk was administered to human volunteers, the strain showed only limited persistence in feces and could not be recovered in 67% of the subjects after 7 days of the last dosage.^75^ The same results were obtained when a milk formula containing the strain was fed to premature infants.^76^ However, Alander et al.^74^ did recover *L. rhamnosus* GG from colonic biopsies for lengthy periods after administration ceased. Human volunteers were administered with 6 × 10^10^ CFU of *L. rhamnosus* GG twice a day for 12 consecutive days. Cell numbers of *L. rhamnosus* GG in the feces decreased with time after the last bacterial dosage was administered. No cells of strain GG were detected in feces 14 days after the last dosage. However, *L. rhamnosus* GG persisted in biopsies taken from the colonic mucosa for up to 21 days at 7 × 10^4^ CFU/biopsy sample after consumption ceased.^74^ Concluded from these studies, fecal cell counts are not a true reflection of the number of viable cells in the GIT of humans and should be accompanied with intestinal biopsies at sites of colonization.

## Competitive exclusion of enteric pathogens

The term competitive exclusion was first used by Greenberg (1969) to describe the exclusion of *S. typhimurium* from blowfly maggots.^77^ This anti-pathogenic mechanism describes the scenario in which one bacterial species rigorously competes for adhesion to receptors in the GIT. The mechanisms of action used by one bacterial species to exclude another from the GIT differ and may include microbe-microbe interactions mediated by binding to the host mucosal interface at specific attachment sites, the secretion of antimicrobial substances and competition for available nutrients.^14,15,17,18^

**Figure 1.**
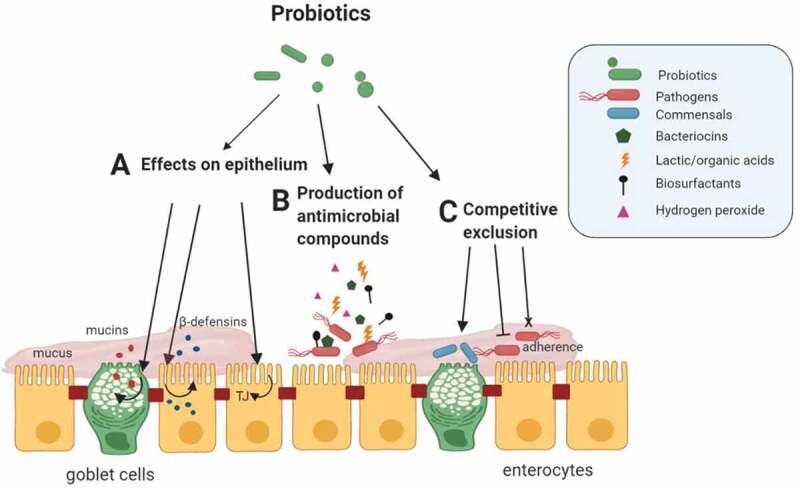
Probiotic mechanisms of action against enteric pathogens in the GIT. Probiotics can affect epithelial barrier integrity by numerous mechanisms. These include: **A**. direct effects on the intestinal epithelial cells (IECs). Probiotics can increase the secretion of mucin glycoproteins by goblet cells that assemble into a thick mucus layer. Probiotics can augment the secretion of antimicrobial proteins (defensins) by IECs that help to eliminate commensals or pathogens that penetrate the mucus layer. Probiotics can enhance the stability of intercellular junctional complexes (tight junctions (TJ)), which decreases the intercellular permeability of IECs to pathogens and other antigens. **B**. Most probiotics can inhibit enteric pathogens via the production of antimicrobial substances such as bacteriocins. **C**. Probiotics can compete with commensals and enteric pathogens for adhesion sites in the mucus layer or IECs, thereby preventing harmful colonization and enhancing barrier function. Probiotics can alter the natural gut microbiota composition and/or gene expression, enhancing barrier integrity through the commensal microbiota. Figure created in biorender (http://biorender.io)

For enteropathogens to initiate infection, they have to cross the intestinal mucosal barrier before colonizing the GIT.^78^ Once pathogens have penetrated the mucus layer overlying the intestinal epithelium, they attach to binding sites on epithelial cells.^79^ Attachment is followed by intestinal colonization and infection.^80^ Probiotics with adhesion capabilities protect the gut against enteric infections by preventing the attachment of pathogens Figure 1. Results from *in vitro* studies using human or animal mucosal material have demonstrated the effect of probiotic LAB on the competitive exclusion of pathogens.^25,48,81–83^ *Lactobacillus rhamnosus* GG has excellent adhesion properties and prevented the internalization of enterohemorrhagic *E. coli* (EHEC) in human intestinal cell lines.^81^ Enteric pathogens, such as EHEC, use mannose-sensitive type 1 fimbriae to attach to oligosaccharide residues of glycoproteins or glycolipids on the surface of intestinal epithelial cells (IECs).^84^ Probiotic strains of lactobacilli and bifidobacteria attach to the same receptor sites and exclude pathogens from binding to the GIT.^85,86^ Some probiotic strains have specific adhesion proteins on their cell surface that bind to carbohydrate moieties in the mucous layer, such as the mannose-specific adherence mechanism of *L. plantarum*.^13,87,88^ In some cases, competitive exclusion may be as simple as steric hindrance.^88^ An overview of studies that analyzed the effect of probiotic LAB surface proteins on adhesion and competitive exclusion using mutant analysis is provided in Table 3. One example of a specific adhesion protein involved in competitive exclusion adhesion-receptor interactions in the GIT is the *L. plantarum* mannose-specific adhesion (Msa) protein.^87^ A spontaneously mutated strain of the probiotic *L. plantarum* 299 v, thought to be affected in the *msa* gene, was unable to inhibit the attachment of EHEC to HT-29 epithelial cells compared to the wild-type.^91^ This suggested that Msa-containing probiotic strains could effectively exclude several other, if not all, type 1 fimbriated enteropathogens. Recently, Van Zyl et al.^37^ demonstrated the involvement of the mucus-adhesion protein (*mapA*) of *L. plantarum* 423 in competitive exclusion of *Listeria monocytogenes* EGDe *in vivo*, using gene knockout analysis and BLI. The *mapA* negative mutant strain of *L. plantarum* 423 was unable to exclude *L. monocytogenes* EGDe.^37^

**Table 3.** Predicted function and mutant phenotypes of probiotic LAB cell surface adhesion genes

Strain	Gene	Predicted function	Mutant phenotype
*L. plantarum* WCFS1 ^89^	*srtA*	Sortase	Reduced mannose-specific binding; competitive ability in murine GIT not affected
*L. plantarum* WCFS1 ^89^	*msa*	Mannose-specific adhesin	Reduced mannose-specific binding
*L. plantarum* WCFS1 ^90^	*lp_2940*	Sortase-dependent cell wall protein	Reduced persistence in murine GIT
*L. plantarum* 299 v ^91^	*msa*	Mannose-specific adhesin	Reduced capability to prevent adherence of EHEC to HT-29 cells
*L. plantarum 423* ^37^	*mapA*	Mucus – adhesion protein (MapA)	Reduced capability to exclude *Listeria monocytogenes* EGDe from the GIT of mice
*L. acidophilus* NCFM ^92^	*mub*	Mucus-binding protein (MUB)	Reduced binding to human Caco-2 cells
*L. acidophilus* NCFM ^92^	*slpA*	S-layer protein	Reduced binding to human Caco-2 cells
*L. salivarus* UCC18 ^93^	*srtA*	Sortase	Reduced binding to human Caco-2 and HT-29 cells
*L. salivarus* UCC18 ^93^	*lspA*	Large surface protein (LSP), putative MUB	Reduced binding to human Caco-2 and HT-29 cells
*L. salivarus* UCC18 ^93^	*lspB*	LSP, putative MUB	Binding to human Caco-2 and HT-29 cells not affected
*L. reuteri* 100–23 ^94^	*lsp*	LSP	Reduced persistence in murine GIT
*L. johnsonii* NCC533 ^21^	*LJ1476*	Transpeptidase Sortase	Colonization dynamics similar to that of wild-type
*E. mundtii* ST4SA ^37^	*srtA*	Sortase-dependent cell wall protein	Reduced capability to exclude *L. monocytogenes* EGDe from the GIT of mice
*E. mundtii* ST4SA ^37^	*srtC*	Sortase-dependent cell wall protein	Reduced capability to exclude *L. monocytogenes* EGDe from the GIT of mice

Another example of a putative competitive exclusion factor is the collagen-binding protein of *L. fermentum*. Heinemann et al.^95^ characterized the collagen surface-binding protein of *L. fermentum* RC-14, which inhibited the adherence of *E. faecalis* 1131. Other studies have demonstrated the role of surface layer (S-layer) extracts in the prevention of pathogens from attaching to, and thus colonizing, IECs.^96,97^ Chen et al.^96^ showed that S-layer proteins anchored on the cell surface of *Lactobacillus crispatus* ZJ001 were responsible for competitive exclusion of *S. typhimurium* and EHEC. Similar results were recorded by Johnson-Henry et al.^97^ The authors showed that S-layer protein extracts from *Lactobacillus helveticus* R0052 inhibited the adhesion of *E. coli* O157:H7 to Caco-2 cells. S-layer proteins are highly hydrophobic and it was suggested that pathogen adherence inhibition was mediated by hydrophobic group interactions as opposed to adhesion-receptor interactions.

Previous studies suggested that sortase-dependent cell surface proteins (SDPs) play a crucial role in probiotic-host interactions, adherence and colonization.^98–102^ Several SDPs have been identified with a role in *in vitro* and *in vivo* adhesion to intestinal cells, including mucus-binding cell surface proteins Table 3. In Gram-positive bacteria, sortases decorate the cell surface with a diverse array of proteins by covalently joining them to the cell wall (Sortase A) or by polymerizing proteins to construct complex multi-subunit pilin structures (Sortase C) on the cell surface.^99^ Sortases are characterized as cysteine transpeptidases that join SDPs containing a specific cell wall sorting signal (CWSS) to an amino group located on the cell surface.^100^ Sortase A enzymes anchor proteins that contain a CWSS with a LPXTG (where X donates any amino acid) C-terminal motif to the cell surface.^99^ The LPXTG motif is recognized by the SrtA enzyme, which breaks the threonine and glycine peptide bond and then covalently links the threonine residue to the amino group of the pentaglycine bacterial cell wall cross bridge.^93,101^ Sortase C proteins catalyze a similar transpeptidation reaction, but recognize a QVPTGV sorting motif to construct pili that promote microbial adhesion.^102^ Using mutant analysis coupled with *in vivo* BLI, a recent study showed that *E. mundtii* ST4SA sortase mutants (*srtA* and *srtC*) had a reduced ability to exclude *L. monocytogenes* EGDe from the GIT of mice compared to the wild-type derivative.^37^

Several strains of lactobacilli and bifidobacteria inhibit, displace, and adhere to the same enterocyte layer as, enteropathogenic *Salmonella choleraesuis* serovar Typhimurium .^103^ This indicates that the probiotic strains have the ability to effectively displace the pathogen after pathogenic colonization of the gut has occurred instead of being effective only when administered in a preventative manner. To gain a competitive advantage, the probiotics can thus modify the gut environment by producing inhibitory compounds, lowering pH levels and competing for nutrients.^64^ *Lactobacillus* species such as *L. acidophilus* and *L. plantarum* have the ability to utilize complex carbohydrates such as fructans.^104,105^ Similarly, bifidobacteria are capable of metabolizing various plant dietary fibers using several depolymerizing enzymes.^105^ Utilizing carbohydrate sources other than those used by enteropathogenic bacteria enable probiotic bacteria to widen their areas of colonization in the GIT and inhibit pathogens.

## Production of antimicrobial compounds

Antimicrobial compounds, produced by probiotic bacteria, can exert direct antimicrobial action toward competing enteropathogens that may lead to the prevention of pathogenic colonization of the GIT Figure 1b.

### Bacteriocins

Bacteriocins are ribosomally produced antimicrobial peptides that differ in terms of their size (2–10 kDa) and mechanisms of action (for a review, see ref. 108). The production of bacteriocins by probiotic bacteria (usually LAB) is a key mechanism of action used to inhibit pathogens in the GIT. Bacteriocins usually only inhibit specific species, often those closely related to the producer.^106,107^ Some bacteriocins are reported to have a much broader spectrum of antimicrobial activity.^107–111^ Bacteriocins such as nisin, produced by *Lc. lactis*, plantaricin from *L. plantarum* and lacticin B from *L. acidophilus* are active against food-borne enteropathogens such as *Listeria, Clostridium, Bacillus*, methicillin-resistant *Staphylococcus aureus* and vancomycin-resistant enterococci (VRE).^14–119^ Bacteriocins may have a bacteriostatic or direct bactericidal effect on pathogens, thus limiting the ability of the cells to colonize the gut. The associated antimicrobial activities of bacteriocins allow bacteriocin-producing probiotic strains to gain a competitive advantage within the complex GI environment.^120^

Bacteriocins adhere to microbial cells and penetrate phospholipid membranes due to their small size and variations in hydrophobic and hydrophilic properties.^121^ The general mechanisms of bacteriocin-mediated pathogen killing include the induction of cytoplasmic membrane permeabilization of sensitive bacteria that leads to cell leakages, inhibition of DNA and RNA synthesis and/or cell wall protein-synthesis.^117,122,123^ For instance, nisin acts by forming a complex with the cell membrane lipid II precursor, followed by the aggregation and incorporation of peptides to form discrete pores in the bacterial cell membrane.^124^ A unique bacteriocin, bifidocin B, produced by *Bifidobacterium bifidum* NCFB, is active against several Gram-positive bacteria, including *Listeria, Enterococcus, Bacillus* and *Lactobacillus*, but shows no activity toward several other Gram-positive and Gram-negative bacteria.^125^ The difference in activity between strains is related to the ability of Gram-negative bacteria to resist adsorption of bifidocin B, due to their cell wall composition.^126^ However, several bacteriocins, such as mutacins (A-D), nisins (A and Z), lacticins (A164 and BH5), bacteriocins E 50–52 and OR7 are active against medically important Gram-negative organisms such as *Campylobacter, Helicobacter, Haemophilus, Neisseria* and *Salmonella* spp.^127–132^ In another study, a bacteriocin produced by *Bacillus amyloliquefaciens* RX7 showed broad-spectrum antibacterial, as well as antifungal, activity and inhibited the growth of *Candida albicans*, the causative agent of cutaneous candidiasis in humans.^133^ While the mode of action of bacteriocins against Gram-positive bacteria has been studied in depth, the direct mechanism of action of bacteriocins against Gram-negative bacteria is poorly understood.^132,134^ Tiwari et al.^134^ demonstrated the ability of bacteriocins enterocin E50-52, pediocin PA-1 and its hybrid peptides, EP and PE, to induce the efflux of intracellular ATP and to dissipate the cellular transmembrane potential of *E. coli* O157:H7 and *S. enterica* serovar Enteritidis 20E1090. Bacteriocins are mostly cationic peptides, and this characteristic enables electrostatic interactions with the negatively charged head groups of bacterial phospholipid.^135^ This is followed by insertion into the planar lipid bilayer or liposome membranes, leading to the formation of transient channels, leakage of cellular contents and subsequent cell death.^136^

In addition to *in vitro* studies, several *in vivo* studies have demonstrated the inhibitory effect of purified bacteriocins and probiotic bacteriocin-producing strains in infectious animal models. Simonova et al.^137^ observed that feeding rabbits with bacteriocin-producing *Enterococcus faecium* CCM7420 and its partially purified bacteriocin significantly reduced *Staphylococci* spp. cell numbers in the cecum thus protecting the animals against infection. Other studies found that the *E. faecium* EK13, enterocin A producing strain reduced *Salmonella* cell numbers in gnotobiotic Japanese quails and reduced the colonization of pathogenic *Staphylococcus* in the digestive tract of rabbits.^138,139^ The capacity of human-isolated nisin- and pediocin-producing LAB to reduce the intestinal colonization of VRE in mice was demonstrated for the first time by Millette et al.^38^ Amyloliquecidin and penisin, produced by *B. amyloliquefaciens* and *Paenibacillus* sp. strain A3, respectively, significantly reduced methicillin resistant *S. aureus* (MRSA) infection levels in mice.^140,141^ Svetoch et al.^142^ reported a significant reduction of *Salmonella enteritidis* in broilers after oral administration of the *E. faecium* E 50–52 bacteriocin. Corr et al.^35^ demonstrated that feeding mice with the *Lactobacillus salivarus* UCC11 bacteriocin Abp118-producing strain reduced *L. monocytogenes* cell numbers in the liver and spleen. A similar reduction in cell numbers of the same pathogen in the GIT of mice was observed when the animals were pre-treated with probiotic strains *L. plantarum* 423 and *E. mundtii* ST4SA, producing bacteriocins plantaricin 423 and mundticin ST, respectively.^36^ Using gene knockout and reverse genetic analysis, the same authors confirmed bacteriocin production and adhesion proteins as mechanisms for the anti-listerial activity.^37^

Several studies have demonstrated the topical application of bacteriocins to treat skin infections, mastitis and oral infections.^140–147^ Despite their powerful anti-infective therapeutic potential and a large selection of isolated and characterized bacteriocins, these peptides have not yet entered into clinical use.^148–150^ This is likely due to various production difficulties.^149,151^ However, progress in preclinical studies of several bacteriocins has proven promising. Several bacteriocins have been through different stages of preclinical development, targeting multi-drug resistant bacteria as well as cystic fibrosis.^149^ These include the bacteriocins, NVB302 and NVB333 (both produced by *Actinoplanes liquriae* NCIMB41362), mutacin 1140 (produced by *Steptococcus mutans* JH1000), NAI-107 (produced by *Microbispora corallina*) and Moli1901 (produced by *Streptomyces cinnamoneum*).^149^

It is also important to consider that not all potential or developed probiotic strains that show *in vitro* antimicrobial activity against enteropathogens will be active *in vivo*. For example, despite the fact that a *Lactobacillus* sp. strain adhered to the jejunum and ileum of gnotobiotic pigs after oral administration and that the strain showed *in vitro* activity against enteropathogenic *E. coli* (EPEC), the strain failed to prevent EPEC colonization in the GIT of infected animals.^152^ Similar results were observed when *L. casei* subsp. *casei* failed to prevent the intestinal colonization of EPEC in the GIT of gnotobiotic or conventional piglets when the LAB strain was administered in a preventative setup.^153^ Nevertheless, most probiotics use their bacteriocins to effectively interact with enteropathogens through either bacteriostatic or bactericidal activities. In doing so, they prevent pathogenic colonization of the host GIT and subsequent occurrence of disease.

### Bacteriocin-like inhibitory substances

Bacteriocin-like inhibitory substances (BLIS) have a broader spectrum of antimicrobial activity. Many of these compounds are not fully characterized or do not share characteristics typical of bacteriocins.^12^ The antimicrobial activities are not related to the production of lactic acid, other organic acids, or hydrogen peroxide.^12,154^ *Lactobacillus rhamnosus* GG secretes an antimicrobial substance with inhibitory activity against *Clostridium* spp., *Staphylococcus* spp., *Enterobacteriaceae, Streptococcus* spp., *Bacteriodes* spp., and *Pseudomonas* spp.^155^ This low molecular weight (LMW) substance is characterized as heat-stable, distinct from lactic and acetic acids, and closely resembles a microcin that is normally produced by *Enterobacteriaeae* spp. These characteristics suggest that it could be a BLIS.^12^ Similar substances with molecular weights and broad activity spectrums uncharacteristic of bacteriocins are produced by other lactobacilli, including strains of *L. acidophilus* and *L. delbrueckii*, their bactericidal affects are related to neither lactic acid nor hydrogen peroxide.^156,157^ Other studies have identified bacteriocin-like antimicrobial substances produced by several *Bifidobacterium* strains with broad spectrums of activity against both Gram-positive and Gram-negative pathogens such as *L. monocytogenes, Salmonella* spp. and *E. coli* spp.^158–160^

### Organic acids

An additional mechanism of pathogen displacement in the gut employed by probiotic bacteria is their ability to make the intestinal environment less suitable for pathogen growth. Probiotic LAB and commensal microbiota ferment carbohydrates in the GIT that lead to the production of metabolites such as acetic, formic, succinic and lactic acids, rendering the intestinal environment acidic and inhibiting the growth of bacterial pathogens.^161^ Organic acids, in particular lactic and acetic acid, repress the growth of many pathogenic bacteria in the GIT.^12,15,64^ The undissociated form of lactic acid functions as a permeabilizer of the Gram-negative bacterial outer cell membrane, after which it dissociates inside the bacterial cytoplasm following entry.^162^ The bacterial killing activity is exerted by lowering the intracellular pH level, through the accumulation of ionized forms of the organic acid and other antimicrobial compounds inside the cytoplasm.^163^

De Keersmaecker et al.^164^ demonstrated that the strong inhibitory effects of *L. rhamnosus* GG against *S. typhimurium* was due to lactic acid production. Lehto and Salminen^165^ demonstrated the potential role of lactic acid in the ability of *Lactobacillus* strain GG to prevent the invasion of Caco-2 cells by *S. enterica* serovar Typhimurium. The authors suggested a pH-dependent mechanism after they observed that inhibition of the pathogen was eliminated when the LAB culture was set to pH 7. In another study, the growth and expression of the HilA and InvF virulence factors by *Salmonella* were affected by lactic acid.^166^

The inhibition of *E. coli* O157:H7 by different *Lactococcus* and *Lactobacillus* strains was attributed to the production of lactic acid and low pH.^167,168^ The growth of *Helicobacter pylori* was inhibited by different *Lactobacillus* and *Bifidobacterium* strains including *L. acidophilus, L. bulgaricus* and *Bifidobacterium bifidus*.^169,170^ These effects were linked to the production of lactic, acetic and hydrochloric acid. In another study, the growth of four species of known enteropathogens, *H. pylori, Campylobacter jejuni, Campylobacter coli* and *C. difficile* was inhibited by *Lactobacillus* strains isolated from the human GIT, probably due to the production of organic acids.^171^ Based on these studies, it is reasonable to suggest that the production of organic acids by probiotics in the GIT makes the intestinal environment less favorable for their competitors and decreases the risk of enteric infections by pathogens.

### Hydrogen peroxide

In addition to lactic acid and bacteriocin production, hydrogen peroxide (H_2_O_2_) production by commensal or probiotic LAB may be an important antimicrobial mechanism against pathogens.^172^ Hydrogen peroxide may cause reduced pathogen virulence, reduced pathogen invasion of epithelial cells or death of intestinal pathogens after epithelial intracellular diffusion which alters gene transcription and signal transduction.^173,174^ Several H_2_O_2_-producing bacterial species with probiotic properties have been isolated, such as *B. bifidum*, the *Lactobacillus johnsonii* NCC 533 gut isolate, a *L. delbrueckii* subsp. *bulgaricus* yogurt isolate and normal microflora vaginal isolates such as *L. crispatus* and *L. gasseri*.^174–181^

The ability of *L. johnsonii* NCC533 to generate up to millimolar quantities of H_2_O_2_ under aerobic conditions has been demonstrated.^176^ The authors demonstrated the antimicrobial role of *L. johnsonii* NCC533 produced H_2_O_2_ against *S. enterica* serovar Typhimurium *in vitro*, and proposed that *L. johnsonii* NCC533 H_2_O_2_-production could contribute to protection against the pathogen *in vivo*. Other studies have shown that H_2_O_2_-producing *L. crispatus* F117 and *Lactobacillus paracasei* strains (F2 and F28) inhibited the growth of *S. aureus in vitro*.^181,182^ The beneficial role of H_2_O_2_-producing probiotic LAB that form part of the vaginal microflora of healthy women has been studied extensively.^180–183^ Previous studies have reported that women carrying H_2_O_2_-producing lactobacilli are less likely to develop bacterial vaginosis.^180,183^

### Siderophores

Iron is an essential micronutrient that plays a central role in the metabolism and proliferation of most gut microbes, including commensal bacteria and gut pathogens.^184^ Siderophores are LMW, organic, high-affinity iron-chelating compounds produced by microorganisms such as bacteria and fungi.^185^ These compounds inhibit the growth, proliferation and persistence of competing microbes by depriving them of iron. In doing so, siderophore-producing bacteria sequester free iron available in their environment that is essential to other microorganisms. For example, the growth of *Lc. lactis, C. difficile* and *Clostridium perfringens* was inhibited in the GIT by iron-binding *Bifidobacterium* strains that produce siderophores.^186^ Growth and adhesion of enteropathogenic *S. typhimurium* N15 and EHEC to IECs were inhibited by *B. pseudolongum* PV8-2 and *Bifidobacterium kashiwanohense* PV20-2 with high iron sequestration properties.^187^

### Biosurfactants

The production of biosurfactants by some LAB is another mechanism that can interfere with pathogen growth in the GIT. Biosurfactants are a group of compounds with surface and emulsifying activities used in many different biomedical applications.^188,189^ Several LAB strains have been isolated that produce either cell-bound or secreted biosurfactants with antibacterial, antiviral and antifungal properties.^188–193^ Biosurfactants cause permeabilization of cells by effecting changes that disrupt or lyse the physical cell membrane structure.^194^ The use of biosurfactant-producing lactobacilli in the prevention of urogenital tract infections is of considerable interest.^188^ These organisms are believed to compete with urogenital bacterial pathogens and yeast for adhesion sites on epithelial cells and control their growth by the production of biosurfactants.^195–197^ In another study, *L. casei* MRTL3 that produces a bacteriocin and a biosurfactant, inhibited a broad range of pathogens, including *L. monocytogenes, S. aureus, Shigella flexneri* and *Pseudomonas aeruginosa*.^198^

### Compounds inhibiting pathogen adhesion to intestinal cells

Adhesion to intestinal cells and subsequent colonization by enteropathogens is regarded a prerequisite for virulence.^199^ Probiotic bacteria produce compounds that do not have a direct bactericidal effect, but contribute to the normal anti-infectious activities of the GIT by inhibiting the binding of pathogenic bacteria to the mucosal surface. Fujiwara et al.^200^ purified and characterized a novel proteinaceous compound in culture supernatants of *B. longum* SBT2928, termed BIF, that inhibits the adhesion of enterotoxigenic *E. coli* Pb176 (ETEC) to human HCT-8 IECs. The authors demonstrated that BIF blocks the binding of the ETEC Pb176 colonization factor antigen (CFA) II adhesive factor to gangliotetraosylceramide (bacterial binding structure) receptors on the intestinal cell surface, thereby preventing ETEC Pb176 colonization.^200^ Two *Bifidobacterium* strains, CA1 and F9, isolated from the GIT of infants produce a LMW, lipophilic, antibacterial compound that inhibits the adhesion of several pathogenic bacteria, including *S. typhimurium* SL1344 and *E. coli* C1845.^201^

## Stabilization of intestinal epithelial barrier

**Figure 2.**
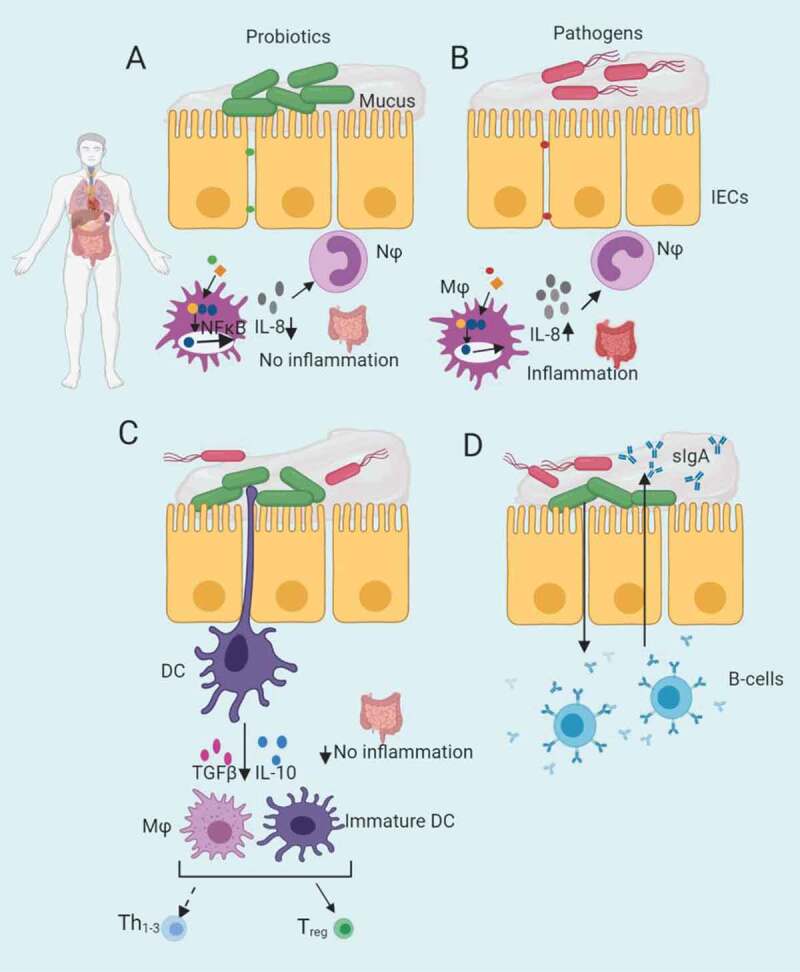
Mucosal immunomodulation by probiotics in the presence of enteric pathogens. **A**. Down-regulation by probiotic bacteria of pro-inflammatory cytokine (IL-8) secretion in the GIT. Probiotic bacteria (or their products) may dampen an innate immune response by inhibiting the NF-_Ƙ_B inflammatory signaling pathway and influencing the production of IL-8 and subsequent recruitment of inflammatory immune cells to sites of intestinal injury. **B**. Activation of NF-_Ƙ_B signaling pathway by enteric pathogens, resulting in severe inflammation of intestinal epithelium tissue. **C**. Probiotic signaling of dendritic cells (DCs) to stimulate the secretion of anti-inflammatory cytokines such as IL-10 in response to an intestinal infection. **D**. Probiotics can augment the levels of IgA-secreting plasma cells in the lamina propria and promote the transcytosis of secretory IgA (sIgA) across the epithelial cell layer and secretion into the luminal mucus layer, preventing and limiting bacterial penetration of host tissues. IECs, intestinal epithelial cells; IL-8, interleukin 8; IL-10, interleukin 10; MФ, macrophage; NФ, neutrophil; NF-_Ƙ_B, nuclear factor-kappa B. TGFβ, transforming growth factor-β; Th_1-3_, T helper cells; T_reg_, regulatory T cells. Figure created in biorender (http://biorender.io)

The GI epithelium consists of a uni-layer of cells covered by a mucus layer that is constantly exposed to the luminal contents and various enteric bacteria.^78,202^ The intestinal epithelial barrier consists of the mucus layer, the intestinal cells and the gut innate immune system.^202^ This GI barrier functions as a key defense mechanism required to maintain epithelial integrity and to prevent infection by pathogens and excessive inflammation. Stabilization and maintenance of this barrier is thus of utmost importance to the host. Important defense mechanisms of the intestinal barrier against unwelcome intrusion of harmful antigens include the mucosal layer (mucin production), intercellular junctional complexes (tight and adherence junctions) and the secretion of antimicrobial peptides (such as defensins) and immunoglobulin A (IgA).^202–205^ Disruption of this barrier function can lead to inappropriate inflammatory responses due to invasion of the submucosa by bacteria or food antigens, which may result in intestinal disorders such as inflammatory bowel disease (IBD) and ulcerative colitis.^203,206,207^ Consumption of colonizing or non-colonizing probiotics can enhance barrier integrity which helps to protect the intestinal epithelium against enteric pathogens and chronic inflammation by direct effects on the epithelium (e.g. increasing mucin expression by goblet cells), modulation of the immune system and by direct effects on commensal and pathogenic bacteria (e.g. antimicrobial peptides and competition for adherence) Figures 1 and 2.

Intestinal epithelium cells are overlaid with a protective inner and outer mucus layer that limits bacterial movement and acts as a dynamic defense barrier against enteropathogens and other potentially harmful antigens.^204^ For enteropathogens to colonize the intestine, they have to penetrate the mucus layer before they reach the intestinal epithelium.^208^ Mucins are the major macromolecular constituents of the epithelial mucus layer and are produced by specialized goblet cells in the intestinal tract.^209^ Probiotics are able to inhibit pathogen adherence to IECs by promoting the secretion of intestinal mucins and defensins Figure 1a. Several *Lactobacillus* species have been shown to increase the expression of specific mucin genes in human intestinal Caco-2 and HT29 cells, thus preventing the adherence and internalization of pathogenic *E. coli*.^91,210,211^ The adherence of EPEC was inhibited by *L. plantarum* 299 v-mediated increase in expression of the MUC2 and MUC3 mucins.^91,211^ Rats administered with VSL3 (pre- and probiotic mixture) for 7 consecutive days showed a 60-fold increase in MUC2 expression and an associated increase in mucin production.^212^ Therefore, increased mucus production mediated by probiotic bacteria *in vivo* may be a key mechanism in their interactions with enteropathogens to prevent infections and to improve intestinal barrier function.

## Co-aggregation

Probiotic bacteria can prevent enteropathogenic adherence and intestinal colonization by co-aggregating with pathogens.^213–215^ In this process, probiotic bacteria interact closely with pathogens, allowing them the opportunity to release their anti-pathogenic substances in proximity to the pathogens. Probiotic LAB can form multi-cellular aggregates that are crucial for colonization of the oral cavity, the urogenital tract and the GIT.^37,213,215–217^ The ability of probiotic cells to co-aggregate is characterized by the clumping of cells that are genetically distinct, whereas auto-aggregation involves cells of the same strain.^218,219^ Auto- and co-aggregation have been reported for various *Lactobacillus* species, including *L. plantarum, L. reuteri, L. gasseri, L. crispatus* and *L. coryniformis*.^219,220^ Several studies have shown that the auto- and co-aggregation abilities of probiotic cells enhances their colonization and may enable the formation of a barrier to prevent colonization by pathogens.^213–215,218^ *Lactobacillus plantarum* strains (S1, A and B) co-aggregate with selected food-borne pathogens including *S. typhimurium* and *L. monocytogenes*.^218^ *Lactobacillus plantarum* S1 co-aggregated best with EHEC at 41.5%, *L. plantarum* A co-aggregated with *S*. Typhimurium at 40.5% and *L. plantarum* B co-aggregated with *L. monocytogenes* at 37.4%.^218^ This is a clear indication that the ability of LAB strains to bind to the food-borne pathogens is not restricted to one species or a single strain. In another study, the adherence of ETEC to porcine enterocytes was affected by co-aggregation of the pathogen with selected *Lactobacillus* spp., including *L. fermentum, L. salivarius* and *L. delbrueckii*. ^221^

The co-aggregation ability of probiotic LAB is generally related to a great diversity of properties among cell surface adherence proteins.^222,223^ Kos et al.^215^ demonstrated that differences in the hydrophobicity and hydrophilicity of the structural cell surface of *L. acidophilus* M9, *L. plantarum* L4 and *E. faecium* L3 may be responsible for the abilities of the strain to co-aggregate. In other examples, proteins involved in the maintenance of cell shape including the S-layer protein CbsA of *L. crispatus* JCM 5810, the DEAD box helicase AggH of *L. reuteri* 1063 and Apf of *L. gasseri* 4B2 were all responsible for the mediation of auto-aggregation.^224–226^ This suggests that the co-aggregation phenomenon of probiotic LAB may be a secondary activity of cell surface components involving random interactions with other surface components. Schachtsiek et al.^219^ described the role of a *Lactobacillus coryniformis* DSM 20001 surface protein encoded by a *cpf* gene (co/aggregation-promoting factor) in the ability of the LAB strain to co-aggregate with *E. coli* K-88, *C. coli* and *C. jejuni*. The auto- and co-aggregation ability of *L. acidophilus* M92 was shown to be mediated by proteinaceous surface layer (S-layer) components, approximated at 45 kDA in size.^215^

Co-aggregation of probiotic and pathogenic bacteria is also mediated via the attachment of probiotic cells to fimbriae expressed on the cell surface of pathogens.^213^ This makes sense, as several studies have shown that probiotic LAB can prevent enteropathogenic binding to intestinal epithelial cells by attaching to the same carbohydrate receptor sites as the pathogens.^14,18,85,86^ The attachment of probiotic cells to the surface of pathogenic cells is dependent on the specific type of fimbriae expressed by the pathogen.^213^ The expression of fimbriae by pathogens is important in colonization of the GIT, the vagina and perineum.^84,196,213,227^ For example, *E. coli* that express type I fimbriae are most commonly associated with urinary tract infections.^227^ Mizuno et al.^220^ presented *E.coli* fimbriae and lipopolysaccharide (LPS) as the essential mediators of the co-aggregation of *L. casei* NBRC 3831 with *E. coli* K-12. Spencer and Chesson^221^ showed that selected strains of lactobacilli co-aggregate with enterotoxigenic *E. coli* expressing K88 fimbriae, but not with a K88-negative knockout mutant strain.

## Inhibition of flagella motility

Flagella are known to play an important role as a virulence factor in many bacterial pathogens.^228^ Flagella allow pathogenic bacteria to respond to attractant and repellent gradients and are crucial for attachment to, and invasion of, eukaryotic cells.^229–232^ Foodborne pathogens such as *S. enterica* serovar Typhimurium require actively rotating flagella to rapidly contact and to efficiently penetrate GI epithelial cells. *Salmonella* Typhimurium remained noninvasive in infected mice when treated with a potent antibody that inhibits flagellum-based motility.^233^ Probiotic bacteria can impair the flagella motility of enteropathogens, thus preventing pathogenic colonization of the gut.

Líeven-Le Moal et al.^234^ demonstrated that antidiarrhoeic *L. acidophilus* LB and its secreted products inhibited the entry of *S. enterica* serovar Typhimurium into human intestinal Caco-2 cells by disrupting the swimming motility of the diarrhea-associated enteropathogen. The authors showed that *L. acidophilus* LB secretes a heat stable LMW product that causes rapid depolarization of the *S*. Typhimurium SL1344 cytoplasmic membrane. The inhibitory activity did not affect bacterial viability or flagellum expression. The transient impairment of the swimming motility of *S*. Typhimurium SL1344 leads to a delay in the pathogen’s capacity to induce F-actin membrane remodeling and thus entry into intestinal Caco-2 cells. In another study, levels of translocated *Salmonella* were dramatically lower in mice orally infected with *S*. Typhimurium when treated with the cell free supernatant of live probiotic lactobacilli, compared to that of untreated mice.^235^ It is possible that the difference between cell numbers of treated and untreated groups is due to a delay in pathogen translocation across the intestinal epithelial barrier caused by the inhibition of *Salmonella* swimming motility. This in turn, exposed the pathogenic cells for extended periods to host defenses in the intestinal lumen that includes antimicrobial products from both the host cells and the microbiota.

## Immune system modulation

It is well known that probiotic bacteria can exert regulatory effects on host innate and adaptive immune responses.^236^ These bacteria have the ability to modulate the functions of dendritic cells (DCs), monocytes/macrophages, and T and B lymphocytes, which enhances phagocytosis of invading gut pathogens.^237,238^ By stimulation of the host immune responses (specific and nonspecific), probiotic bacteria can displace pathogens in the GIT and prevent intestinal diseases.^238–241^

Probiotic bacteria can interact with pathogens in the gut by antagonizing inflammatory responses induced by the gut pathogens.^236^ Inflammation allows pathogens to flourish at the expense of the natural microbiota and host intestinal health. Probiotic bacteria are able to trigger an anti-inflammatory response from the innate immune system by signaling DCs to secrete anti-inflammatory cytokines such as interleukin 10 (IL-10) Figure 2c.^242,243^ They can also elicit a decrease in pro-inflammatory cytokines during inflammation.^237^ Down-regulation of pro-inflammatory cytokine secretion from immune cells occurs as a result of probiotic bacterial interference with inflammatory signaling pathways such as nuclear factor-kappa B (NF-_Ƙ_B) and mitogen-activated protein kinases (MAPK) Figure 2a.^244,245^ The activation of these signaling pathways leads to the secretion of pro-inflammatory cytokines that can severely damage the intestinal epithelial barrier. NF-_Ƙ_B and MAPK signaling pathways are activated by enteric pathogens to stimulate the secretion of pro-inflammatory cytokines (e.g. IL-8), that lead to the recruitment of inflammatory immune cells (e.g. neutrophils) to the infected area resulting in severe inflammation, tissue damage and disease Figure 2b.^244,245^ Several studies have identified probiotic strains with the ability to suppress pro-inflammatory cytokine production to avoid pathogen-induced inflammation at infection sites.^236,245–247^ A study by Finamore et al.^246^ reported that *Lactobacillus amylovorus* DSM 16698 protected IECs against the pro-inflammatory response induced by ETEC K88 through the suppression of pro-inflammatory cytokines IL-8 and IL-1ß. Another study demonstrated the ability of *L. casei* OLL2768 to suppress the ETEC-induced pro-inflammatory response by inhibition of NF-_Ƙ_B and MAPK pathways that reduced pro-inflammatory cytokine levels.^247^

Probiotics also play a role in the stimulation and production of antibodies in the gut, particularly immunoglobulin A (IgA) Figure 2d.^236,237^ Antibodies released in the intestinal lumen can inhibit pathogen adherence to IECs by interfering with adhesive cell receptors on the pathogen’s cell membrane. Previous studies have indicated that *Saccharomyces boulardii* and *L. rhamnosus* GG increased secretory IgA levels or immunoglobulin-secreting cell levels in the GIT.^248,249^ Other studies have reported that oral administration of probiotic lactobacilli increased IgA levels in children suffering from diarrhea, thereby shortening the duration of symptoms.^237,250–252^ Several probiotic strains can also modulate the host immune mechanisms by influencing phagocytosis of enteric pathogens by host immune phagocytic cells such as macrophages.^14,18,64,248,251,253,254^ The inhibition of enteropathogenic *P. aeruginosa* and *L. monocytogenes* in mice by a strain of *L. casei* correlates to an increase in abundance of macrophages.^255^ Furthermore, probiotic bacteria can affect phagocytotic cell activities not only in clinical situations but also in healthy subjects.^256–258^

## Conclusion

Elucidating the mechanisms of action of probiotic microorganisms is a difficult task given the complex nature of the human GI ecosystem. Probiotic mechanisms of action employed against enteric pathogens are diverse, heterogeneous and may be strain specific. This suggests that the mechanism(s) of action of one specific probiotic strain against a particular disease or pathogen cannot be generalized since different strains evoke different responses in the host. Thus, the health benefits conferred by one strain are not applicable to another strain, even within the same species. Understanding the full potential of probiotics for therapeutic or prophylactic applications against GI diseases requires thorough investigations of probiotic-host and probiotic-pathogen interactions. Enhanced understanding of these interactions will enable the identification of true probiotics to target specific enteric diseases. While a number of recent *in vivo* studies have demonstrated the mechanistic basis behind observed probiotic effects at the molecular level, greater emphasis is warranted in this area of probiotic research. Unraveling the intricacies of probiotic-host and probiotic-pathogen interactions will improve the *in vitro* selection of the best probiotics based on key properties such as bacteriocin production and adhesion genes. Moreover, an efficient probiotic should exhibit stimulation of the hosts’ immune system and ultimately, must have demonstrable beneficial health effects on the host. It is clear that the demonstration of key antimicrobial and protective probiotic mechanisms *in vivo* will allow for industry and consumers to choose scientifically validated probiotics for the prevention or treatment of various health problems.
